# Supplementary material for: Genome‐Wide Diversity in Lowland and Highland Maize Landraces From Southern South America: Population Genetics Insights to Assist Conservation
Source: Evol Appl. 2024 Dec 1;17(12):e70047. doi: 10.1111/eva.70047 (PMC11609054; doi:10.1111/eva.70047)
Supplement: Supplementary file 1 — Figure S1. Supporting Information of the principal component discriminant analysis (DAPC) performed with Adegenet in R (Jombart, 2008) and shown in Figure 2C. A Variance explained by PCA (principal component analysis). B) Values of BIC (Bayesian information criterion) versus number of clusters. C) DAPC cross validation. D) Contingency table of the K = 3 DAPC (x‐axis: DAPC groups, y‐axis: maize classification, size of squares: number of individuals). E) Density graph for K = 2. F) Contingency table of the K = 2 DAPC (x‐axis: DAPC groups, y‐axis: maize classification, size of squares: number of individuals). HNWA: Highland maize of Northwestern Argentina. LNWA: Lowland maize of Western Argentina. PNWA: Popcorn of Northwestern Argentina. FNEA: Floury maize of Northeastern Argentina. PNEA: Popcorn of Northeastern Argentina. Total number of individuals: 87. [file EVA-17-e70047-s008.pdf]

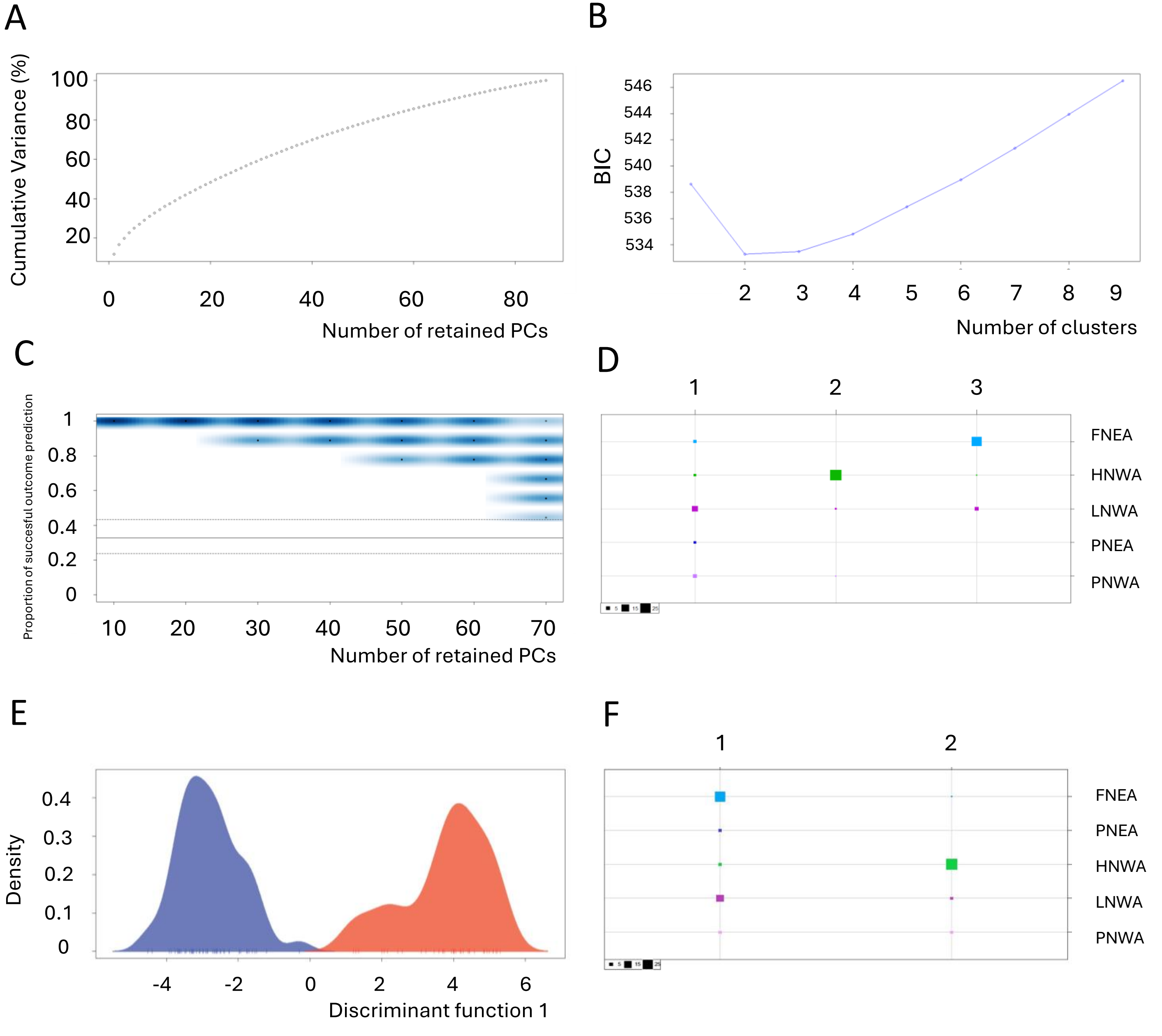

Supplementary Figure 1. Supplementary data of the Principal Component Discriminant Analysis (DAPC) performed with Adegenet in R (Jombart, 2008) and shown in Figure 2C. A) Variance explained by PCA (Principal Component Analysis). B) Values of BIC (Bayesian information criterion) versus number of clusters. C) DAPC cross validation. D) Contingency table of the K=3 DAPC (x-axis: DAPC groups, y-axis: maize classification, size of squares: number of individuals). E) Density graph for K=2. F) Contingency table of the K=2 DAPC (x-axis: DAPC groups, y-axis: maize classification, size of squares: number of individuals). HNWA: Highland maize of Northwestern Argentina. LNWA: Lowland maize of Western Argentina. PNWA: Popcorn of Northwestern Argentina. FNEA: Floury maize of Northeastern Argentina. PNEA: Popcorn of Northeastern Argentina. Total number of individuals: 87.
