# Supplementary material for: Genome‐Wide Diversity in Lowland and Highland Maize Landraces From Southern South America: Population Genetics Insights to Assist Conservation
Source: Evol Appl. 2024 Dec 1;17(12):e70047. doi: 10.1111/eva.70047 (PMC11609054; doi:10.1111/eva.70047)
Supplement: Supplementary file 2 — Figure S2. Hardy–Weinberg equilibrium obtained with VCFtools (Danecek et al. 2011) in (A) floury maize of Northeastern Argentina (FNEA), (B) highland maize of Northwestern Argentina (HNWA) and (C) all individuals employing the χ2 test. Upper panel: Excess heterozygotes. Lower panel: Heterozygotes in default. The plots show the p‐values versus SNP genomic positions. Red dots indicate statistically significant excess or defect heterozygotes (p‐value < 1.42e‐5;p‐values corrected for multiple testing by the Bonferroni test). [file EVA-17-e70047-s009.pdf]

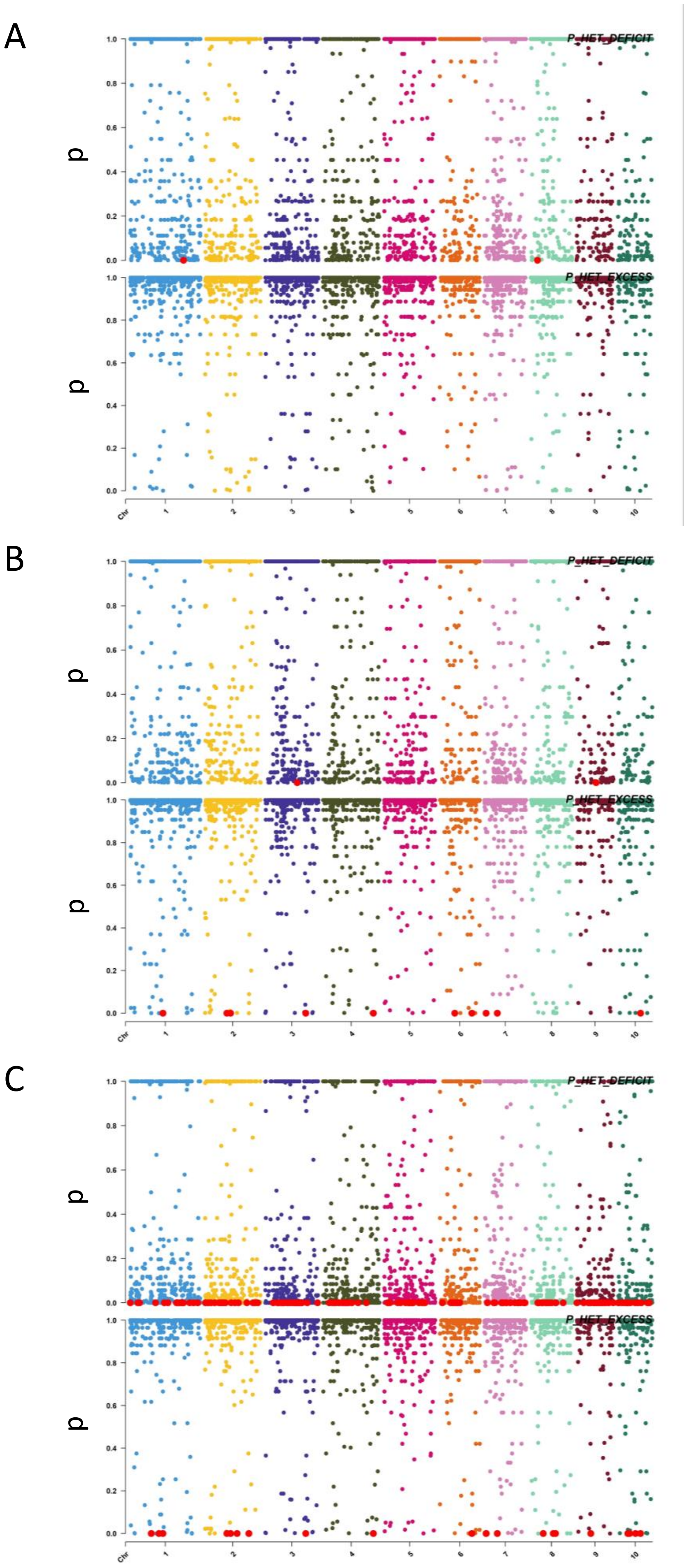

Supplementary figure 2. Hardy-Weinberg equilibrium obtained with VCFtools (Danecek et al., 2011) in (A) Floury maize of Northeastern Argentina (FNEA), (B) Highland maize of Northwestern Argentina (HNWA) and (C) all individuals employing the χ^2 test. Upper panel: excess heterozygotes. Lower panel: heterozygotes in default. The plots show the p-values versus SNP genomic positions. Red dots indicate statistically significant excess or defect heterozygotes (p-value < 1.42e-5; p-values corrected for multiple testing by the Bonferroni test).
