# Supplementary material for: Genome‐Wide Diversity in Lowland and Highland Maize Landraces From Southern South America: Population Genetics Insights to Assist Conservation
Source: Evol Appl. 2024 Dec 1;17(12):e70047. doi: 10.1111/eva.70047 (PMC11609054; doi:10.1111/eva.70047)
Supplement: Supplementary file 4 — Figure S4. Close‐ups of habitat suitability modelling of highland northwestern maize (HNWA) performed with MaxEnt (Phillips et al. 2006) employing (A) altitude and historical climate data, and future climate scenario models (B) CNRM‐CM6‐1 (Voldoire et al. 2019) and (C) MRI‐ESM2‐0 (Yukimoto et al. 2019) for the period 2081–2100 and the SSP1‐2.6 CO2 emission scenario. [file EVA-17-e70047-s001.pdf]

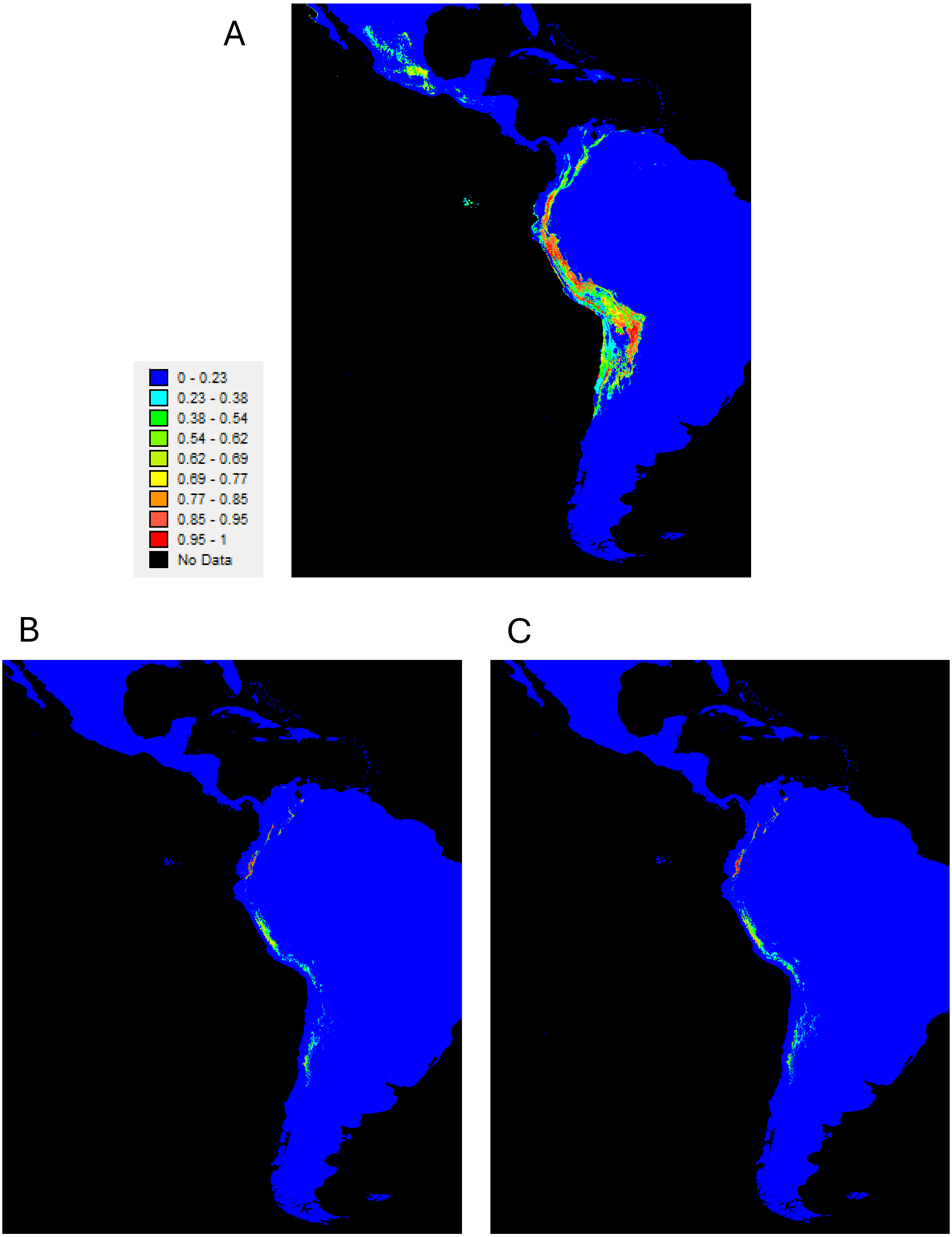

Supplementary Figure 4. Close-ups of habitat suitability modelling of Highland Northwestern maize (HNWA) performed with MaxEnt (Phillips et al., 2006) employing (A) altitude and historical climate data, and future climate scenario models (B) CNRM-CM6-1 (Voldoire et al., 2019) and (C) MRI-ESM2-0 (Yukimoto et al., 2019) for the period 2081-2100 and the SSP1-2.6 CO_2 emission scenario.
