# Supplementary material for: Genome‐Wide Diversity in Lowland and Highland Maize Landraces From Southern South America: Population Genetics Insights to Assist Conservation
Source: Evol Appl. 2024 Dec 1;17(12):e70047. doi: 10.1111/eva.70047 (PMC11609054; doi:10.1111/eva.70047)
Supplement: Supplementary file 5 — Figure S5. Jackknife of regularised training gain for MaxEnt (Phillips et al. 2006) model of (A) floury maize of Northeastern Argentina (FNEA) and for (B) highland maize of Northwestern Argentina (HNWA) employing historical bioclimatic variables and altitudes from Worldclim (https://www.worldclim.org/data/cmip6/cmip6climate.html). Green: Without variable. Blue: With only variable. Red: All variables. (C) Definition of the variables employed in the analyses. [file EVA-17-e70047-s007.pdf]

A

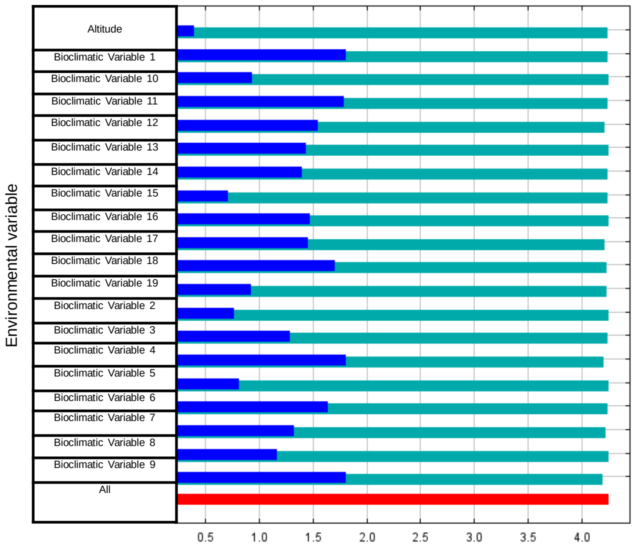

B

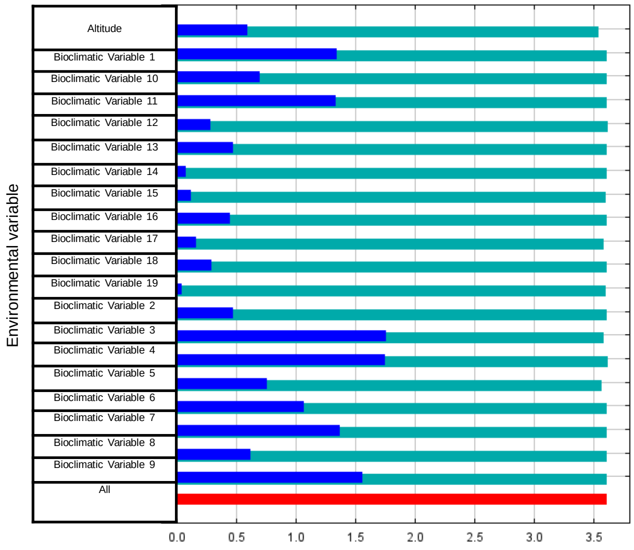

C

Bioclimatic Variable	Definition
Bioclimatic Variable 1	Annual Mean Temperature
Bioclimatic Variable 2	Mean Diurnal Range (Mean of monthly (max temp - min temp))
Bioclimatic Variable 3	Isothermality (Bioclimatic variable 2/Bioclimatic variable 7) (×100)
Bioclimatic Variable 4	Temperature Seasonality (standard deviation ×100)
Bioclimatic Variable 5	Max Temperature of Warmest Month
Bioclimatic Variable 6	Min Temperature of Coldest Month
Bioclimatic Variable 7	Temperature Annual Range (Bioclimatic variable 5-Bioclimatic variable 6)
Bioclimatic Variable 8	Mean Temperature of Wettest Quarter
Bioclimatic Variable 9	Mean Temperature of Driest Quarter
Bioclimatic Variable 10	Mean Temperature of Warmest Quarter
Bioclimatic Variable 11	Mean Temperature of Coldest Quarter
Bioclimatic Variable 12	Annual Precipitation
Bioclimatic Variable 13	Precipitation of Wettest Month
Bioclimatic Variable 14	Precipitation of Driest Month
Bioclimatic Variable 15	Precipitation Seasonality (Coefficient of Variation)
Bioclimatic Variable 16	Precipitation of Wettest Quarter
Bioclimatic Variable 17	Precipitation of Driest Quarter
Bioclimatic Variable 18	Precipitation of Warmest Quarter
Bioclimatic Variable 19	Precipitation of Coldest Quarter
Altitude	Height above sea level

Supplementary figure 5. Jacknife of regularised training gain for MaxEnt (Phillips et al., 2006) model of (A) Floury maize of Northeastern Argentina (FNEA) and for (B) Highland maize of Northwestern Argentina (HNWA) employing historical bioclimatic variables and altitudes from Worldclim (https://www.worldclim.org/data/cmip6/cmip6climate.html). Green: without variable. Blue: with only variable. Red: all variables. (C) Definition of the variables employed in the analyses.
